# Good character at school: positive classroom behavior mediates the link between character strengths and school achievement

**DOI:** 10.3389/fpsyg.2015.00610

**Published:** 2015-05-15

**Authors:** Lisa Wagner, Willibald Ruch

**Affiliations:** ^1^Personality and Assessment, Department of Psychology, University of Zurich, Zurich, Switzerland; ^2^Distance Learning University Switzerland, Brig, Switzerland

**Keywords:** character strengths, virtues, VIA classification, positive education, adolescents, positive psychology, school achievement, character

## Abstract

Character strengths have been found to be substantially related to children’s and adolescents’ well-being. Initial evidence suggests that they also matter for school success (e.g., Weber and Ruch, 2012). The present set of two studies aimed at replicating and extending these findings in two different age groups, primary school students (*N* = 179; mean age = 11.6 years) and secondary school students (*N* = 199; mean age = 14.4 years). The students completed the VIA-Youth (Values in Action Inventory of Strengths for Youth), a self-report measure of the 24 character strengths in the VIA classification. Their teachers rated the students’ positive behavior in the classroom. Additionally, school achievement was assessed: For the primary school students (Study 1), teachers rated the students’ overall school achievement and for the secondary school students (Study 2), we used their grades as a measure of school achievement. We found that several character strengths were associated with both positive classroom behavior and school achievement. Across both samples, school achievement was correlated with love of learning, perseverance, zest, gratitude, hope, and perspective. The strongest correlations with positive classroom behavior were found for perseverance, self-regulation, prudence, social intelligence, and hope. For both samples, there were indirect effects of some of the character strengths on school achievement through teacher-rated positive classroom behavior. The converging findings from the two samples support the notion that character strengths contribute to positive classroom behavior, which in turn enhances school achievement. Results are discussed in terms of their implications for future research and for school interventions based on character strengths.

## Introduction

School achievement is substantially linked with later life outcomes (for an overview, see e.g., Duckworth and Allred, 2012). Behavior in the classroom was found to predict later academic achievement (Alvidrez and Weinstein, 1999) and also important life outcomes in education and the labor market, even beyond the influence of achievement in standardized tests (Segal, 2013). Therefore, studying the influence of non-intellectual aspects on educational outcomes has a long tradition. Also specifically studying good character or positive personality traits had already been addressed by early educational psychologists (e.g., Smith, 1967), but had then been neglected for a long period of time. Only with the advent of positive psychology, it has received revived interest.

Within positive psychology, education is seen as an important area of application. Seligman et al. (2009) defined positive education as “education for both traditional skills and for happiness” (p. 263). Inherent in positive education is the idea that good character, positive behaviors at school and academic achievement are not only aims of education, but also closely intertwined. However, little is known empirically about this interplay. The importance of good character in education has recently been emphasized both in scientific and popular literature (e.g., Tough, 2012; Linkins et al., 2015) and researchers from neighboring disciplines (e.g., Hokanson and Karlson, 2013) have also called for studying the role of character strengths in education.

In the present paper, we take a closer look at the link between students’ character strengths and school achievement and investigate the mediating role of positive behavior in the classroom further. More specifically, we examine whether character strengths facilitate positive classroom behaviors, which in turn facilitate attaining higher grades. Character strengths are not only expressed in thoughts and feelings, but importantly, also in behaviors (Peterson and Seligman, 2004). We expected that a number of strengths are very helpful for schoolwork and are thus robustly related to positive behaviors in the classroom, as the teachers can observe it. Such positive classroom behaviors, e.g., actively in class or showing motivation to learn, should ultimately contribute to school achievement. We aim to provide a better insight into which aspects of good character are reliably linked with school achievement and positive classroom behavior and for which of the character strengths the link between them and school achievement is mediated by positive classroom behavior. To achieve this aim, we use two samples representing primary and secondary education, and perform analyses on the level of single character strengths. This detailed level of analysis may be especially interesting when relating the results to programs that emphasize the cultivation of certain character strengths.

### Character Strengths in Children and Adolescents

Peterson and Seligman’s (2004) classification allows studying good character and its contribution to positive development in a comprehensive way. The VIA classification describes 24 character strengths, that are organized under six, more abstract, virtues (wisdom and knowledge, courage, humanity, justice, temperance, and transcendence) and are seen as ways to reach these virtues. Character strengths are seen as inherently valuable, but also contribute to positive outcomes (Peterson and Seligman, 2004). Character strengths can be seen as the components of a good character, and are described as the inner determinants of a good life, complemented by external determinants (such as safety, education, and health; cf. Peterson, 2006). Since the development of the VIA classification and the Values in Action Inventory of Strengths for Youth (VIA-Youth; Park and Peterson, 2006), which reliably assesses the 24 character strengths in children and adolescents between 10 and 17 years, a number studies in different cultures have revealed substantial links between character strengths and subjective well-being of children and adolescents (Van Eeden et al., 2008; Gillham et al., 2011; Weber et al., 2013; Ruch et al., 2014b).

### Character Strengths and School Achievement

A large number of studies have examined the links between broad personality traits and academic achievement. Meta-analyses (e.g., Poropat, 2009, 2014a) reveal that conscientiousness is the strongest correlate, whereas the links between extraversion, neuroticism, agreeableness, and openness/intellect with academic achievement have been rather weak and inconsistent. These links are largely independent of intelligence (Poropat, 2009) and personality traits have even been found to be equally strong predictors of academic achievement than intelligence when they were self-rated, and even stronger predictors when they were other-rated (Poropat, 2014a). In the available meta-analyses on the relationship between self-rated personality traits and academic achievement, almost all included studies examined students in tertiary education (Poropat, 2009) or they even focused only on postsecondary education (e.g., Richardson et al., 2012; McAbee and Oswald, 2013). A recent meta-analysis (Poropat, 2014b), however, examined the predictive validity of adult-rated personality traits for academic achievement in primary education and found that conscientiousness and openness had the strongest correlations with measures of school achievement. Still, it has to be noted that we know a lot more about how personality, especially when it is self-rated, is related to academic achievement, and about what might be relevant mechanisms behind it, in young adults than we know about these relationships in children and adolescents. And, although authors have speculated that the relationship between personality and academic achievement is attributable to “positive traits that naturally promote academic learning” (Medford and McGeown, 2012, p. 787), those studies did not investigate narrower, positively valued personality traits specifically.

Some aspects of good character have been studied in relation to school achievement. Duckworth and colleagues (Duckworth and Seligman, 2005; Duckworth et al., 2007) demonstrated the relevance of self-regulation and grit for academic achievement beyond measured intelligence. Also other character strengths, such as hope (e.g., Levi et al., 2014), have been shown to relate to academic achievement. In contrast to approaches that consider only some aspects of good character, the VIA classification (Peterson and Seligman, 2004) offers a comprehensive catalogue of character strengths. Weber and Ruch (2012) provided an initial investigation of the role of the 24 character strengths in school. In a sample of 12-year old Swiss school children, they studied the relationship between character strengths, positive experiences at school, teacher-rated positive classroom behavior, and school achievement. A factor representing character strengths of the mind (e.g., love of learning, perseverance, prudence) was related to school achievement, which was operationalized by grades in mathematics and German language. Specific character strengths (e.g., perspective, gratitude, hope, self-regulation, perseverance, love of learning) were higher in those students with improved grades during the course of the school year, than in those with decreased grades. Similarly, in a sample of Israeli adolescents at the beginning of middle school, Shoshani and Slone (2013) found intellectual and temperance strengths to be predictors of grade point average (GPA).

### Character Strengths and Positive Classroom Behavior

Park and Peterson (2006) found moderate convergence between self- and teacher-reported character strengths and argued that certain strengths may be more readily observable in the classroom than others. Especially phasic strengths, which can only be displayed when the situation demands it (e.g., bravery), may be more difficult to observe than tonic strengths, which can be displayed in any situation (e.g., kindness; cf. Peterson and Seligman, 2004). Even though the frequency might vary, character strengths are expressed in overt behavior, so they should also contribute to positive behavior in the classroom. In particular, temperance strengths (e.g., prudence, self-regulation) should be helpful to regulate feelings, thoughts, and behaviors in a way that matches the expectations and norms in the classroom (e.g., showing good conduct). Other strengths, such as social intelligence should be helpful to manage conflict and relationships with classmates successfully, and thus be related to social aspects of positive classroom behavior (e.g., being cooperative). Finally, strengths that were found to be related to school achievement, such as perseverance and love of learning, should also be associated with achievement-related aspects of positive classroom behavior (e.g., working autonomously).

Empirically, Shoshani and Slone (2013) found interpersonal strengths to be related with social functioning at school, which was rated by the teachers, and thus might represent positive social classroom behavior. Weber and Ruch (2012) have studied the relationship with character strengths and positive classroom behavior using their Classroom Behavior Rating Scale (CBRS), assessing both achievement-related and social classroom behavior. In a multiple regression analysis, about 25% of the variance in teacher-rated positive classroom behavior was explained by the 24 character strengths. Perseverance, prudence, and love of learning showed the most substantial correlations with teacher-rated positive classroom behavior.

### Positive Classroom Behavior as a Mediator of the Relationship between Character Strengths and School Achievement

High scores in good character do not automatically and directly lead to high levels of school achievement, but they will predispose students to show a set of more proximate behaviors, which in turn predispose for higher grades later on. Thus, if certain character strengths are identified as being related to school achievement, it is of course interesting to examine potential mechanisms involved. One likely candidate for explaining this link is positive behavior in the classroom, since the grading of students is largely depending on the behaviors that teachers can observe in the classroom, and especially such behaviors that they value (e.g., showing a high motivation to learn, adhering to classroom rules). Weber and Ruch (2012) used a latent variable representing classroom-relevant character strengths (love of learning, perseverance, and prudence) showed an indirect effect on school achievement mediated by positive classroom behavior. After adding the mediator to the model, there was no direct effect of character strengths on school achievement, which is in line which a full mediation by positive classroom behavior.

### Aims of the Present Study

The presented studies strongly suggest that character strengths are indeed important resources at school, supporting school achievement either directly, or also indirectly via the display of positive behavior in the classroom. There is, however, a need to further investigate these relationships to examine their robustness and also potential moderators. In addition, these initial studies also have several limitations. First, many included only students in rather narrow age ranges and from one level of education. While the study by Weber and Ruch (2012) does include a broader range of level of education, it may be somewhat limited by the fact that teachers only knew their students for about three months when they were rating their positive classroom behavior. Second, in most studies, character strengths were analyzed only on the factor level–four factors in Shoshani and Slone (2013) and two factors in Weber and Ruch (2012)–and it is difficult to draw conclusions on the level of specific strengths based on these results. Doing so may be especially interesting when evaluating the results in light of programs or interventions that build on the cultivation of certain strengths (e.g., grit/perseverance or self-regulation).

The present studies aimed at replicating the findings by Weber and Ruch (2012) and extending them by including students in different school types (Study 1: primary school, Study 2: secondary school) and a broader range of school grades beyond grades in mathematics and German language (Study 2). We will also investigate for each of the character strengths individually whether the potential link with school achievement is mediated by positive classroom behavior. In doing so, the present study will add to the knowledge on the role of positive traits for positive behavior and achievement at school.

While none of the 24 character strengths should be detrimental for positive classroom behavior or school achievement, certain strengths should be more important than others. Based on theoretical assumptions and previous empirical findings, we expect certain character strengths to be related to positive classroom behavior and school achievement most strongly. These nine character strengths are: perseverance, self-regulation, prudence, love of learning, hope, gratitude, perspective, teamwork, and social intelligence.

Firstly, we expect *perseverance* to be robustly related to the educational outcomes measured. Students high in perseverance are characterized by “voluntary continuation of a goal-directed action in spite of obstacles, difficulties, and discouragements” (Peterson and Seligman, 2004, p. 229). Such behaviors are highly advantageous in a school environment, in which challenging goals are presented and sustained efforts despite obstacles are needed to accomplish them. Since perseverant individuals enjoy finishing tasks, the completion of, e.g., an assignment may be particularly rewarding for them. Thus, perseverance can be seen as a helpful resource both for displaying positive behavior in the classroom (e.g., behaving diligently) and for school achievement, because perseverant students will work persistently on tasks and homework, even when it is difficult, and thus might be more successful in consequence. Secondly, *self-regulation* is expected to be associated with educational outcomes. Self-regulation helps to control own feelings and appetites. Thus, it is helpful to avoid obstacles and reach goals or meet expectations of others (cf. Peterson and Seligman, 2004). At school, it is often demanded and expected to control one’s own feelings and to conform to what is expected (cf. Ivcevic and Brackett, 2014). Consequently, self-regulation will likely go along with helpful behaviors and strategies at school, such as managing time well, making plans and sticking to them, and adhere to rules. These positive behaviors will be observable in the classroom and may also contribute to higher grades. Thirdly, we expect *prudence* to be related mostly to positive behavior in the classroom, but also to school achievement. Students high in prudence that are particularly careful in their choices (cf. Peterson and Seligman, 2004) are less likely to do things in the classroom that fall outside the teachers’ and classmates’ expectation. Consequently, they are more likely to comply with rules and work toward achieving what is expected of them. Being prudent may also help to avoid interpersonal problems, and thus lead to better relationships with teachers and classmates, which then may be supportive of school achievement. Recently, Ruch et al. (2014a) established that there are different types of class clowns, but each of them was low in prudence. When we assume that class clowns would score quite low on teacher-rated positive classroom behavior and that their characteristics do not fit well with what is required in the classroom, this suggests that being prudent might be crucial for displaying positive behavior in the classroom. Fourthly, we expect *love of learning* to be relevant for predicting behavior and success at school. Individuals high in love of learning experience positive emotions when learning new things, and enjoy doing so whenever possible (cf. Peterson and Seligman, 2004). In any case, attending a school will offer opportunities to learn new things on a daily basis. It is likely that the high intrinsic motivation to learn also leads to better learning outcomes, and that the positive emotions associated with learning additionally foster school achievement (cf. Schutz and Lanehart, 2002; Weber et al., 2014). In the initial study by Weber and Ruch (2012), love of learning, perseverance and prudence were among the most important variables in predicting positive classroom behavior and also had an indirect effect on school achievement through positive classroom behavior.

In addition to these four strengths that are assumed to be helpful at school, we also expect *hope* to be related to behavior and achievement at school. Hopeful individuals are not only characterized by believing that a positive future is likely, but also by acting in ways supposed to make desired outcomes (e.g., achieving a good result in an exam) more likely (Peterson and Seligman, 2004). These desired outcomes can be both in relation to positive behavior in the classroom and to thoughts and behaviors that support achievement, but are not directly observable in the classroom (such as favorable attributions, etc.). Earlier studies have also found that hope predicts future academic achievement (e.g., Marques et al., 2011) as well as demonstrated a close link between hope, effort, and school achievement (Levi et al., 2014). Sixthly and seventhly, *perspective* and *gratitude* may also be relevant in the classroom. Students high in the character strength perspective have consistent ways of looking at the topics and the world, which are meaningful to them and also make sense to others (cf. Peterson and Seligman, 2004). On the one hand, expressing and applying such coherent worldviews at school may help solving problems and integrating different perspectives. On the other hand, perspective is also displayed by giving good and wise advice to others, which may foster positive relationships with classmates, and in turn facilitate learning and achievement. Grateful students are highly aware of the positive things in their lives, and are thankful for these (cf. Peterson and Seligman, 2004). One of the mechanisms conceivable is that these students perceive school as a meaningful institution and are more aware than others of the possibilities that good achievement will offer them in the future. In the study by Weber and Ruch (2012), both perspective and gratitude were higher in those students that improved their grades over the course of the school year than in those that had deteriorated grades. Finally, we expect *social intelligence* and *teamwork* to be related to positive classroom behavior. School is an environment characterized by constant interactions with classmates and teachers. Highly social intelligent individuals understand both their own and others’ feelings, and are able to adapt to other’s feelings and expectations (cf. Peterson and Seligman, 2004). Similarly, individuals high in *teamwork* identify with a group of which they are members (e.g., a classroom) and do their share as group members because they fell if is the right thing to do (cf. Peterson and Seligman, 2004) Therefore, social intelligence and teamwork should both be linked with few conflicts, good cooperation, and adherence to expectations and rules in the classroom, which is all reflected in the teacher-rated positive classroom behavior.

## Study 1

In Study 1, we aim at extending the findings by Weber and Ruch (2012), that is, that the association between certain character strengths and school achievement is mediated by positive behavior in the classroom. We investigate this relationship in a sample of primary school students and a sample of homeroom teachers, using a self-report measure of character strengths, and teacher ratings to assess positive classroom behavior and school achievement. Further, we extend previous studies by studying the assumed mediation on the level of single strengths. We expect an indirect effect mediated by positive behavior, and that the strength of this indirect effect varies for different character strengths.

### Method

#### Participants

The sample of students consisted of 179 German-speaking primary school students (48.6% females) attending the fifth or sixth grade. Their mean age was 11.56 years (*SD* = 0.75; ranging from 10 to 13 years). The majority (86.6%) of participants were Swiss citizens (including dual citizens; data from one participant missing). The sample of teachers consisted of nine homeroom teachers (77.8% men) with a mean age of 36.2 years (*SD* = 7.3; ranging from 23 to 45 years). They had been teaching the participating students for an average of 1.4 years (*SD* = 1.0).

#### Instruments

The German adaptation (Ruch et al., 2014b) of the *VIA-Youth* (Park and Peterson, 2006) is a self-report instrument assessing the 24 character strengths uses seven to nine items per scale utilizing a 5-point response format (from 5 = *very much like me* to 1 = *not like me at all*). It consists of 198 items and about one third of the items are reverse coded. A sample item is “Even when my team is losing, I play fair” (fairness). The VIA-Youth proved to be a reliable and valid measure of self-reported character strengths in previous studies (e.g., Park and Peterson, 2006; Ruch et al., 2014b). In this study, most of the 24 VIA-Youth scales yielded satisfactory internal consistencies (i.e., 17 scales had alpha coefficients > 0.70) and only five scales (modesty: α = 0.51, curiosity: α = 0.55, open-mindedness: α = 0.61, fairness: α = 0.62, and prudence: α = 0.63) had alpha coefficients < 0.65. Altogether, the internal consistency coefficients of the 24 VIA-Youth scales yielded a median of α = 0.72. Means for each of the five factors (leadership, temperance, intellectual, transcendence, and other-directed strengths) were computed (cf. Weber et al., 2013; Ruch et al., 2014b).

The *CBRS* (Weber and Ruch, 2012) assesses teacher ratings of their perceptions of positive behavior in the classroom. The 10 items use a 5-point response scale (from 1 = “not like him/her at all” to 5 = “very much like him/her”) and include both positive achievement-related behavior (e.g., “behaves diligently”) and positive social behavior (e.g., “shows appropriate conflict management”). In the present study, the scale yielded a high internal consistency (α = 0.89).

A teacher rating was also used to assess *school achievement*. Homeroom teachers were instructed to rate the “overall school achievement” (taking into account performance in all subjects) on a scale ranging from 1 = “unsatisfactory” to 7 = “excellent.”

#### Procedure

Data for this study were collected in nine classrooms of three primary schools in German-speaking Switzerland. After obtaining approval by the ethical committee of the philosophical faculty at the University of Zurich, schools were contacted and asked to participate. Participation was voluntary and none of the students or teachers was paid for their participation. All students and a parent or legal guardian gave active consent to participate. A trained psychologist instructed the students and they completed the self-report questionnaires (as part of a larger questionnaire study) in the classroom setting. The teachers completed the rating form. Students received written feedback on their individual rank order of character strengths and were provided with more detailed information on the meaning of the character strengths in the VIA classification. The presented data were collected as a part of a larger project. Whereas Weber et al. (2014) focused on the relationships between character strengths, school-related positive affect, and school achievement in students attending different school types, the present study uses a subset of the sample used by Weber et al. (2014), i.e., only primary school students, and it investigates the relationships between character strengths, positive classroom behavior, and school achievement.

#### Data Analysis

**FIGURE 1:**
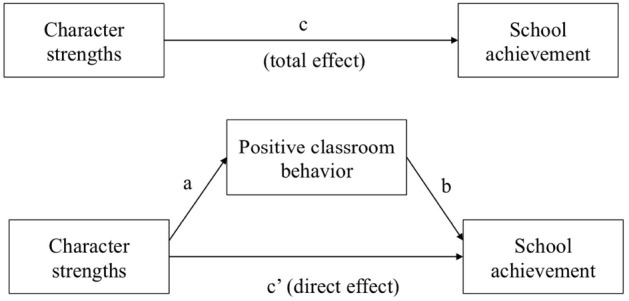
**The mediating role of positive classroom behavior in explaining the relation between 24 character strengths and school achievement; indirect effects tested separately for each of the character strengths**.

The nine character strengths expected to show the most substantial associations were spread out to four of the five higher-order factors (cf. Ruch et al., 2014b) and five (Ruch and Proyer, 2015) or six (Peterson and Seligman, 2004) of the six ubiquitous virtues, so we decided to analyze the data on the level of single strengths instead of on the level of factors. For an initial examination, we computed descriptive statistics of the self-rated character strengths. Furthermore, internal consistency coefficients (Cronbach’s alpha) and correlations with students’ age and sex were computed. Since we observed some age and sex differences in our variables of interest, we decided to control for the influence of these demographic variables in the further analyses. As a second step, we computed partial correlations between character strengths, positive classroom behavior, and school achievement, while controlling for students’ age and sex. In addition, we computed hierarchical multiple regression analyses (controlling for age and sex in the first step) and tested the incremental effect (change in adjusted *R*^2^) of the 24 character strengths entered in the second step. As a final step, we conducted mediation analyses to test the direct and indirect effects of character strengths on school success. The mediation model is displayed in Figure 1. Mediation analyses were conducted with the help of an SPSS macro using bootstrapping with *z* = 5,000 resamples to compute 99.6% confidence intervals (corrected for multiple comparisons) for the indirect effects (Hayes, 2013). Standardized values of all variables were used in the mediation analyses.

### Results

#### Preliminary Analyses and Relationships between Character Strengths, Positive Classroom Behavior, and School Achievement

The results of the preliminary analyses are displayed in Table 1. Means for the VIA-Youth ranged between 3.31 (leadership) and 4.13 (gratitude), and were comparable to the means reported in Ruch et al. (2014b). Also in line with previous findings (Park and Peterson, 2006; Ruch et al., 2014b), there were no substantial correlations with age, and scores on kindness and appreciation of beauty and excellence were higher for girls than for boys. School achievement was negatively correlated with age, and girls received higher ratings in positive classroom behavior than boys.

**TABLE 1:** **Means, standard deviations, internal consistency coefficients, correlations with students’ age and sex of all variables, and correlations with positive classroom behavior and overall school achievement**.

**Variables**	***M***	***SD***	**α**	*r*_age_	*r*_sex_	**PCB**	**OSA**
VIA-Youth scales							
Creativity	3.65	0.51	0.65	0.11	–0.19	0.13	0.22*
Curiosity	3.41	0.47	0.55	–0.07	–0.11	0.18	0.15
Open-mindedness	3.50	0.43	0.61	0.17	–0.03	0.18	0.20
Love of learning	3.59	0.60	0.77	–0.03	0.09	0.34*	0.33*
Perspective	3.57	0.52	0.72	0.08	0.13	0.32*	0.40*
Bravery	3.55	0.52	0.70	0.11	0.10	0.07	0.12
Perseverance	3.73	0.53	0.73	0.02	–0.03	0.40*	0.33*
Honesty	3.64	0.55	0.80	0.15	0.17	0.27*	0.26*
Zest	3.71	0.53	0.72	–0.03	–0.04	0.36*	0.24*
Love	4.01	0.49	0.66	–0.01	0.12	0.23*	0.14
Kindness	3.95	0.50	0.76	0.15	0.46*	0.16	0.21
Social intelligence	3.72	0.49	0.71	0.19	0.14	0.31*	0.32*
Teamwork	3.93	0.50	0.76	0.19	0.07	0.25*	0.25*
Fairness	3.51	0.49	0.62	0.08	0.13	0.24*	0.13
Leadership	3.31	0.61	0.77	0.05	–0.11	0.24*	0.35*
Forgiveness	3.90	0.56	0.71	0.01	0.15	0.23*	0.21
Modesty	3.46	0.43	0.51	–0.03	0.09	0.14	0.17
Prudence	3.40	0.51	0.63	0.04	0.01	0.34*	0.31*
Self-regulation	3.52	0.54	0.71	0.10	0.01	0.32*	0.26*
Beauty	3.75	0.63	0.77	0.10	0.35*	0.07	–0.03
Gratitude	4.13	0.45	0.72	–0.01	0.10	0.27*	0.23*
Hope	3.75	0.51	0.72	–0.03	–0.13	0.41*	0.33*
Humor	3.82	0.59	0.74	–0.13	–0.05	0.13	0.29*
Religiousness	3.90	0.80	0.88	0.11	0.01	0.14	0.01
Teacher ratings							
PCB	3.99	0.73	0.89	–0.12	0.27*	0.66*	
OSA	4.77	1.46		–0.22*	0.11		

N = 179. Age: 10–13 years. Sex: 1 = male; 2 = female. VIA-Youth - VIA Inventory of Strengths for Youth; Beauty - appreciation of beauty and excellence; OSA - overall school achievement; PCB - positive classroom behavior (Classroom Behavior Rating Scale). *p < 0.05 (Bonferroni corrected, one-tailed).

As shown in Table 1, 15 of the 24 character strengths were correlated with positive classroom behavior with the numerically highest coefficients being found for hope, perseverance, zest, love of learning, and prudence. Similarly, 14 of the 24 character strengths were related to teacher-rated school achievement. Perspective, leadership, perseverance, love of learning, hope, and prudence yielded the numerically highest coefficients. The significant correlations were exclusively positive. Multiple hierarchical regression analyses revealed that the 24 character strengths when added in a second step (after controlling for age and sex in the first step) explained 19.7% additional variance (adjusted *R*^2^) in positive classroom behavior, *F*_change_(24,152) = 2.99, *p* < 0.001, and 23.9% additional variance in overall school achievement, *F*_change_(24,152) = 3.47, *p* < 0.001.

#### Positive Classroom Behavior as a Mediator of the Relationship between Character Strengths and School Achievement

Table 2 shows the results of the mediation analyses (Hayes, 2013). There were total effects for 14 of the 24 character strengths and for most of these (all except creativity and humor), there were indirect effects (*a* × *b*), which means that the relationship between the character strengths and school achievement was mediated by positive classroom behavior. For perspective and leadership, there was both an indirect and a direct effect. For the remaining character strengths, the results were consistent with a full mediation—there was only an indirect effect and no significant direct effect. Humor was the only character strength that yielded a significant direct effect, but no indirect effect. Thus, the positive relationship between humor and school achievement was not mediated by positive classroom behavior.

**TABLE 2:** **Results of mediation analyses for character strengths as predictors of overall school achievement with positive classroom behavior as mediator (controlling for age and sex)**.

****	****	****	**Total effect**	**Direct effect**	**Mediation by positive classroom behavior**	**Total *R*^2^**
	***a***	***b***	***c***	*c′*	**indirect effect *a* × *b***	
Creativity	0.13	0.65*	0.23*	0.15	0.09	0.48*
Curiosity	0.18	0.66*	0.15	0.03	0.12	0.47*
Open-mindedness	0.18	0.65*	0.21	0.09	0.12	0.47*
Love of learning	0.33*	0.63*	0.35*	0.13	0.22^a^	0.48*
Perspective	0.33*	0.60*	0.40*	0.20*	0.20^a^	0.50*
Bravery	0.07	0.66*	0.12	0.07	0.05	0.47*
Perseverance	0.41*	0.64*	0.35*	0.08	0.26^a^	0.47*
Honesty	0.26*	0.65*	0.26*	0.08	0.17^a^	0.47*
Zest	0.35*	0.67*	0.24*	0.01	0.23^a^	0.46*
Love	0.22*	0.67*	0.14	–0.01	0.15	0.47*
Kindness	0.16	0.65*	0.22	0.12	0.11	0.47*
Social intelligence	0.31*	0.63*	0.32*	0.13	0.19^a^	0.48*
Teamwork	0.25*	0.65*	0.25*	0.09	0.16^a^	0.47*
Fairness	0.24*	0.68*	0.14	–0.03	0.16	0.46*
Leadership	0.24*	0.62*	0.34*	0.20*	0.15^a^	0.50*
Forgiveness	0.23*	0.66*	0.22	0.06	0.15	0.46*
Modesty	0.14	0.66*	0.17	0.08	0.09	0.47*
Prudence	0.35*	0.63*	0.33*	0.11	0.22^a^	0.47*
Self-regulation	0.31*	0.65*	0.26*	0.06	0.20^a^	0.47*
Beauty	0.07	0.68*	–0.03	–0.08	0.05	0.47*
Gratitude	0.26*	0.65*	0.23*	0.06	0.17^a^	0.46*
Hope	0.40*	0.64*	0.33*	0.08	0.26^a^	0.47*
Humor	0.13	0.64*	0.28*	0.20*	0.08	0.50*
Religiousness	0.14	0.68*	0.01	–0.08	0.09	0.47*

N = 179. Beauty - Appreciation of beauty and excellence. a—Direct effect of IV (character strength) on mediator (positive classroom behavior). b—Direct effect of mediator (positive classroom behavior) on DV (school achievement). c—Total effect of IV (character strength) on DV (school achievement). c′—Direct effect of IV (character strength) on DV (school achievement). a × b—Indirect effect of IV (character strength) on DV (school achievement) through proposed mediator (positive classroom behavior). ^a^The 99.6% CI obtained for the indirect effect by bootstrapping did not include 0. z = 5000 bootstrap resamples. *p < 0.05 (Bonferroni corrected, one-tailed).

### Summary of Results and Limitations

Study 1 was primarily designed to replicate previous findings by Weber and Ruch (2012), and to extend these findings by looking at whether positive classroom behavior mediates the link between character strengths and school achievement on the level of single strengths. We found that a large number of character strengths were linked to teacher-reported positive classroom behavior and school achievement, and that many of the relationships with school achievement were fully mediated by positive classroom behavior. Perspective, leadership, and humor (also) showed direct effects on school achievement, independent of positive classroom behavior.

The interpretation of these results is somewhat limited by the fact that the ratings of positive classroom behavior and school achievement were done by only one teacher, and at the same time. In consequence, the two ratings may be somewhat confounded. Also, we only assessed overall school achievement and we do not know how much emphasis the teachers put on academic vs. non-academic subjects, when evaluating the students’ overall school achievement. Even though it can be assumed that these ratings are valid, it would be desirable to obtain the actual grades and ratings of positive classroom behavior that several teachers have agreed on. Especially when studying the relevance of good character in secondary school classrooms, this would be desirable, since students are in touch with a broader group of teachers than they are in primary school. Looking at grades in academic and non-academic subjects separately would also help to better understand what potential mechanisms are involved in the association between character strengths, positive classroom behavior, and school achievement.

## Study 2

Study 2 aims at extending the findings of Study 1 in three ways: (a) by studying students in secondary school, (b) by using a rating system for positive behavior that has been established in schools and reflects the perspective of several teachers, and (c) by studying associations with actual grades in both academic and non-academic subjects. We expect that the results of Study 1 will be replicated in Study 2, although different measures for both positive classroom behavior and school achievement are used.

We expect somewhat lower effect sizes, since previous research has shown that personality traits tend to play a stronger role in predicting achievement on the primary school level than on secondary school level (Poropat, 2009). Similarly, we expect the correlation between positive classroom behavior and school achievement to be somewhat lower, while still substantial. As a consequence, we also expect that there will be fewer character strengths showing an indirect effect on school achievement through positive classroom. More importantly, we expect stronger relationships for grades in academic than for grades in non-academic subjects, since character strengths should support achievement-related behavior especially in those subjects that require sustained effort and that are less dependent of a specific talent, such as musicality.

### Method

#### Participants

The sample consisted of 199 German-speaking secondary school students (53.3% females) attending the seventh to ninth grade. 37.2% of the students attended a secondary school with basic requirements (qualifying them to begin an apprenticeship after graduation) and 62.8% attended a secondary school with augmented requirements (qualifying them to attend to higher education like university after graduation). Their mean age was 14.42 years (*SD* = 1.19; ranging from 12 to 17 years). The majority (76.4%) of participants were Swiss citizens (including dual citizens).

#### Instruments

We used the German version (Ruch et al., 2014b) of the *VIA-Youth* (Park and Peterson, 2006) to assess *self-reported character strengths*. In Study 2, the internal consistency coefficients of the 24 VIA-Youth scales yielded a median of α = 0.78. Only one scale had an alpha coefficient below 0.65 (modesty: α = 0.64) and 22 of the 24 yielded coefficients > 0.70.

The *positive classroom behavior teacher ratings* is a standard used by schools in Switzerland to describe positive behavior in the classroom. In this study, we used ratings of achievement-related (e.g., “works diligently and reliably”) and social behavior (“is considerate toward other students”). The seven items that were rated on a 4-point response scale (from 1 = “inadequate” to 4 = “very good”) showed a high content overlap with the items of the CBRS (Weber and Ruch, 2012). These ratings were given by the respective students’ teachers collectively and discussed during a teacher meeting. We tested the dimensionality of the teacher ratings using principal component analysis. One eigenvalue exceeded unity (eigenvalues were 3.76, 0.85, 0.66, 0.60, 0.45, 0.35, etc.) and this first factor explained 53.7% of the variance. Parallel analysis (Horn, 1965) suggested unidimensionality as well. Corrected item-total correlations ranged from *r* = 0.52 to *r* = 0.71 (mean *r* = 0.62), and the ratings showed a high internal consistency in the present study (α = 0.85). In the analyses, we consequently used a mean score across all seven items.

*School achievement* was operationalized by *students’ grades* that were provided by the schools’ administration offices. Grades were coded on a scale ranging from 1 = “inadequate” to 6 = “very good” (allowing for half points), with all grades of 4 and higher representing an evaluation of satisfactory achievement, and 3.5 and lower describing unsatisfactory achievement. We computed students’ GPAs as an average across all academic subjects (mathematics, German, French, and English language, history, and science; i.e., excluding music, arts, and physical education). We also calculated an average across grades in mathematics and German language (MG), the two grades commonly considered most important, and an average for grades in non-academic subjects (NA; including art, music, and physical education).

#### Procedure

Data for this study were collected in 14 classrooms of four secondary schools in German-speaking Switzerland, which represented two different educational levels. After obtaining approval by the ethical committee of the philosophical faculty at the University of Zurich, schools were contacted and asked to participate. Students and, in case of participating students under the age of 14 years, also a parent or legal guardian gave active consent.

Classroom teachers were instructed on how to oversee the completion of the questionnaire and how to respond to questions. They read standardized instructions to the students who completed the self-report questionnaire (as part of a larger study) in the classroom setting. Students received written feedback on their individual rank order of character strengths and were provided with information on the meaning of the character strengths of the VIA classification. The schools’ administrative offices provided students’ grades (including the teacher ratings on positive classroom behavior) at the end of the school term, which was a couple of weeks after the data collection had taken place.

#### Data Analysis

In preliminary analyses, we computed means and standard deviations for all assessed variables. In addition, internal consistencies (Cronbach’s alpha) and correlations with age, sex, and school level (basic vs. augmented requirements). To address our research questions, we computed partial correlations (controlling for age, sex, and school level) of the 24 character strengths with positive classroom behavior, and three different indicators of school achievement: GPA, an average across grades in mathematics and German language (MG), and an average for grades in non-academic subjects (NA; including art, music, physical education). As a second step, we conducted mediation analyses to test the direct and indirect effect of character strengths on school success as a third step (see Study 1).

### Results

#### Preliminary Analyses and Relationships between Character Strengths, Positive Classroom Behavior, and School Achievement

As shown in Table 3, means for the VIA-Youth ranged between 3.31 (leadership) and 4.19 (gratitude), and were comparable to the means reported in previous studies as well as in Study 1. There were only a few correlations with age, and scores on bravery, kindness, beauty, and religiousness were higher for girls than for boys. Teamwork, modesty, and hope were higher in students attending schools with augmented requirements, whereas religiousness was higher in students attending schools with basic requirements. Positive classroom behavior was positively correlated with age, and GPA was unrelated to age and sex. Both positive classroom behavior and GPA were higher for students attending schools with augmented requirements than for students attending schools with basic requirements. As some of the variables appeared to be affected by participants’ demographics, we controlled for such influences in subsequent analyses.

**TABLE 3:** **Means, standard deviations, internal consistency coefficients, correlations with students’ age and sex of all variables, and partial correlations with positive classroom behavior and overall school achievement (controlling for students’ age, sex, and school level)**.

**Variables**	***M***	**SD**	**α**	*r*_age_	*r*_sex_	*r*_level_	**PCB**	**GPA**	**MG**	**NA**
VIA-Youth scales										
Creativity	3.55	0.58	0.79	–0.01	–0.02	–0.01	–0.06	0.02	0.00	0.07
Curiosity	3.42	0.54	0.73	0.01	0.05	0.05	0.06	0.19	0.18	0.07
Open-mindedness	3.50	0.52	0.77	0.17	–0.05	0.16	0.07	0.11	0.09	0.03
Love of learning	3.40	0.58	0.74	–0.05	0.17	0.06	0.14	0.25*	0.23*	0.04
Perspective	3.72	0.51	0.74	0.20*	0.11	0.15	0.17	0.21*	0.17	0.10
Bravery	3.69	0.56	0.77	0.17	0.28*	0.10	–0.01	0.01	–0.03	0.03
Perseverance	3.65	0.57	0.80	–0.01	–0.03	0.07	0.22*	0.27*	0.23*	0.14
Honesty	3.79	0.55	0.81	0.09	0.13	0.08	0.18	0.14	0.11	0.06
Zest	3.59	0.55	0.77	0.01	–0.05	0.19	0.13	0.22*	0.25*	0.20
Love	4.04	0.59	0.79	0.06	0.14	0.10	0.13	0.17	0.21*	0.12
Kindness	4.00	0.51	0.80	0.08	0.39*	0.04	0.01	0.02	0.02	0.03
Social intelligence	3.83	0.47	0.66	0.21*	0.09	0.19	0.21*	0.17	0.18	0.06
Teamwork	3.94	0.50	0.74	0.15	0.01	0.24*	0.14	0.11	0.14	0.16
Fairness	3.64	0.50	0.71	0.18	0.16	0.21	0.17	0.09	0.06	0.05
Leadership	3.31	0.66	0.84	0.18	–0.01	0.21	0.12	0.12	0.15	0.17
Forgiveness	3.69	0.67	0.80	0.00	–0.12	0.20	0.08	0.25*	0.26*	0.16
Modesty	3.58	0.50	0.64	0.14	0.02	0.25*	0.10	0.04	0.08	–0.05
Prudence	3.45	0.53	0.71	0.04	–0.15	0.15	0.23*	0.22*	0.12	0.00
Self-regulation	3.59	0.58	0.75	0.14	–0.14	0.11	0.24*	0.19	0.20	0.09
Beauty	3.54	0.70	0.80	0.14	0.40*	0.03	0.10	0.05	0.05	0.14
Gratitude	4.19	0.52	0.79	0.01	0.07	0.15	0.11	0.23*	0.20	0.14
Hope	3.92	0.56	0.82	0.25*	–0.06	0.30*	0.24*	0.33*	0.30*	0.18
Humor	4.05	0.61	0.84	0.17	0.15	0.06	–0.08	0.08	0.08	0.02
Religiousness	3.38	1.00	0.89	–0.12	0.33*	–0.31*	–0.02	0.06	0.06	0.13
Teacher ratings, grades										
PCB	3.24	0.37	0.85	0.28*	0.11	0.52*		0.55*	0.39*	0.18
MG	4.56	0.50		–0.15	0.12	0.09				
GPA	4.61	0.44		0.07	0.19	0.31*				
NA	5.06	0.32		–0.17	0.12	0.15				

N = 199. Age: 12–17 years. Sex: 1 = male; 2 = female. School level: 1 = basic requirements; 2 = augmented requirements. VIA-Youth - VIA Inventory of Strengths for Youth; Beauty - appreciation of beauty and excellence; PCB - positive classroom behavior; GPA - grade point average (only academic subjects: mathematics, German, French, and English language, history, science); MG - average for grades in mathematics and German language; NA - grades in non-academic subjects (art, music, physical education). *p < 0.05 (Bonferroni corrected, one-tailed).

Perseverance, social intelligence, prudence, self-regulation, and hope were positively correlated with teacher-rated positive classroom behavior (see Table 3). Notably more character strengths were positively associated with school achievement, as operationalized by the grade average across all academic subjects: Love of learning, perspective, perseverance, zest, forgiveness, prudence, gratitude, and hope. Correlations with the average of grades in mathematics and German language were similar (although non-significant for perspective, prudence and gratitude). None of the 24 character strengths correlated with grades in non-academic subjects (art, music, physical education), with zest yielding the numerically highest correlation coefficient (*r* = 0.20, *p* = 0.004).

Multiple hierarchical regression analyses revealed that the 24 character strengths when added in a second step (after controlling for age, sex, and school level in the first step), explained 7.3% additional variance (adjusted *R*^2^) in positive classroom behavior, *F*_change_(24,170) = 1.92, *p* < 0.01, 14.8% additional variance in GPA, which was computed across all academic subjects, *F*_change_(24,170) = 2.79, *p* < 0.01, and 13.4% additional variance in Grades in mathematics and German language, *F*_change_(24,170) = 2.30, *p* < 0.01. However, the 24 character strengths explained no significant amount of variance in grades in non-academic subjects beyond the influence of age, sex, and school level, *F*_change_(24,170) = 1.45, *p* = 0.09.

#### Positive Classroom Behavior as a Mediator of the Relationship between Character Strengths and School Achievement

To test the direct and indirect effects of character strengths on school achievement (GPA across academic subjects), mediation analyses were conducted using the bootstrapping procedure suggested by Hayes (2013). Figure 1 shows an illustration of the tested mediation model and results are displayed in Table 4.

**TABLE 4:** **Results of mediation analyses for character strengths as predictors of GPA with positive classroom behavior as mediator (controlling for students’ age, sex, and school level)**.

****	****	****	**Total effect**	**Direct effect**	**Mediation by positive classroom behavior**	**Total *R*^2^**
	***a***	***b***	***c***	***c*′**	**Indirect effect *a* × *b***	
Creativity	–0.04	0.56*	0.01	0.04	–0.03	0.39*
Curiosity	0.05	0.54*	0.17	0.14	0.03	0.41*
Open-mindedness	0.06	0.55*	0.10	0.07	0.03	0.39*
Love of learning	0.12	0.52*	0.24*	0.17*	0.06	0.42*
Perspective	0.15	0.53*	0.20*	0.12	0.08	0.40*
Bravery	–0.01	0.55*	0.01	0.02	0.00	0.39*
Perseverance	0.19*	0.51*	0.24*	0.15	0.10^a^	0.41*
Honesty	0.16	0.54*	0.13	0.04	0.08	0.39*
Zest	0.08	0.53*	0.21*	0.15	0.06	0.41*
Love	0.11	0.54*	0.16	0.10	0.06	0.40*
Kindness	0.01	0.55*	0.02	0.02	0.00	0.39*
Social intelligence	0.18*	0.54*	0.16	0.07	0.10^a^	0.39*
Teamwork	0.12	0.55*	0.10	0.03	0.07	0.39*
Fairness	0.15	0.55*	0.09	0.01	0.08	0.39*
Leadership	0.11	0.55*	0.11	0.06	0.06	0.39*
Forgiveness	0.07	0.54*	0.23*	0.20*	0.04	0.43*
Modesty	0.08	0.55*	0.04	–0.01	0.05	0.39*
Prudence	0.20*	0.53*	0.20*	0.10	0.11^a^	0.40*
Self-regulation	0.21*	0.54*	0.18	0.07	0.11^a^	0.39*
Beauty	0.09	0.55*	0.05	0.00	0.05	0.39*
Gratitude	0.09	0.53*	0.21*	0.16	0.05	0.41*
Hope	0.21*	0.50*	0.32*	0.22*	0.10^a^	0.43*
Humor	–0.07	0.56*	0.07	0.11	–0.04	0.40*
Religiousness	–0.02	0.55*	0.06	0.07	–0.01	0.39*

N = 199. Beauty - Appreciation of beauty and excellence. a—Direct effect of IV (character strength) on mediator (positive classroom behavior). b—Direct effect of mediator (positive classroom behavior) on DV (school achievement). c—Total effect of IV (character strength) on DV (school achievement). c′—Direct effect of IV (character strength) on DV (school achievement). a × b—Indirect effect of IV (character strength) on DV (school achievement) through proposed mediator (positive classroom behavior). ^a^The 99.6% CI obtained for the indirect effect by bootstrapping did not include 0. *p < 0.05 (Bonferroni corrected, one-tailed).

As shown in Table 4, eight character strengths yielded total effects on school achievement, as operationalized by GPA (across academic subjects). Hope yielded both a direct effect and an indirect effect through positive classroom behavior, which is in line with a partial mediation. Perseverance and prudence yielded indirect effects without direct effects, which is in line with a full mediation of the relationship by positive classroom behavior, and there was an additional indirect effect for social intelligence and self-regulation. Love of learning and forgiveness yielded only a direct effect, thus their relationship with school achievement was not mediated by positive classroom behavior.

## General Discussion

The present study extends the knowledge on the role of character strengths for positive behavior and achievement at school. We used two different samples to replicate and extend previous findings on the link between primary and secondary school students’ character strengths, positive classroom behavior, and school achievement. Using a sample of primary school students, results of Study 1 showed that hope, perseverance, zest, love of learning, prudence, perspective and self-regulation were most substantially correlated with teacher-rated positive behavior in the classroom. Perspective, leadership, love of learning, perseverance, social intelligence, hope, and prudence yielded the highest correlations with overall school achievement, as rated by the students’ homeroom teachers. For 12 of the 24 character strengths, mediation analyses revealed an indirect effect through positive classroom behavior on school achievement. Using a sample of secondary school students and actual grades, results of Study 2 showed that hope, self-regulation, prudence, perseverance, and social intelligence were related to positive classroom behavior, that eight character strengths were related to GPA across academic grades, and that none of the character strengths was correlated with grades in non-academic subjects. Mediation analyses revealed that the associations with GPA were (partly) mediated by positive classroom behavior for some of the character strengths, but not for others.

There were some striking similarities in the results of both studies. In both studies, perseverance, social intelligence, prudence, self-regulation, and hope were related to positive classroom behavior, and love of learning, perspective, perseverance, zest, prudence, gratitude, and hope were related to school achievement. Compared to typical effect sizes for the relationship between personality traits and academic achievement, the effect sizes that we found for several character strengths are comparable to or exceed those reported for conscientiousness in meta-analyses (cf. Poropat, 2009).

Perseverance, prudence and hope were associated with both positive classroom-behavior and school achievement across the two studies presented here. Social intelligence and self-regulation showed replicable associations across both samples only with positive classroom behavior, but were not related consistently with school achievement. Love of learning, perspective, zest and gratitude showed a replicable association with school achievement, but were not consistently associated with positive classroom behavior. When comparing these results to our expectations, eight of the nine character strengths showed the expected associations with school achievement and/or positive classroom behavior across both studies. The ninth strength, teamwork, only showed associations with both variables in Study 1, but not Study 2. In addition, zest was robustly associated with school achievement. While love of learning is specifically related to positive experience while learning new things, zestful students are generally more vital, alert and energetic (cf. Peterson and Seligman, 2004). Zest is highly related to experiencing positive affective states in general (e.g., Van Eeden et al., 2008), but also at school (Weber et al., 2014). This suggests that being zestful is a helpful resource also for school achievement, e.g., by maintaining high levels of energy when being faced with schoolwork.

All character strengths that yielded indirect effects on school achievement through positive classroom behavior in Study 2 (perseverance, prudence, self-regulation, hope) had also yielded indirect effects in Study 1. Hope additionally yielded a direct effect on school achievement in Study 2. The effects of perseverance and prudence on school achievement were fully mediated by positive classroom behavior in both studies. Perseverance and prudence thus seem to be related to school achievement mostly through mechanisms that are observed and appreciated by the teachers. This seems plausible as both of these strengths are theoretically linked with adherence to rules and conforming with expectations, while controlling impulses and feelings that are repugnant to those. Hope, on the other hand, seems to affect school achievement also through mechanisms that are not captured by teacher-rated positive classroom behavior.

There were also differences between the results of the two studies. Most strikingly, the number of character strengths associated with positive classroom behavior and (potentially as a consequence) the number of character strengths whose effects on school achievement were mediated by positive classroom behavior was much higher in the sample of primary school students (Study 1) than in the sample of secondary school students (Study 2). This cannot be explained by differences in sample sizes, which were minor anyway. Study 2 also showed that there were no relationships with grades in non-academic subjects. It is possible that specific talents (e.g., musicality, sportiness) play a more important role for achievement in such subjects. This result also suggests that character strengths are (at least not only) related to school achievement because “being the nice student” will make the grade in just any subject. It seems rather that character strengths facilitate achievement-related behavior that then may lead to better school achievement. The fact that Study 1 considered teacher ratings of overall school achievement which also included non-academic subjects might also account for a portion of the differences in the results between the two studies.

### Limitations and Future Research

In the two studies, we used slightly different measures of positive classroom behavior and school achievement. While this can be seen as supporting the robustness of the findings, one could also argue that this makes the results less comparable. Indeed, it is difficult to disentangle which of the differences between the results are accounted for by sample characteristics (age, school type) or by differences in the measures. However, especially the measures of positive school behavior showed a high content overlap and teacher ratings of school achievement at primary school level have been shown to be highly related with actual grades (e.g., *r* = 0.88 in Spinath and Spinath, 2005).

The interpretation of our findings is of course also limited by the cross-sectional nature of the study, which does not allow drawing causal conclusions. While in many cases it seems likely that the character strength contributes to school achievement, in other cases also an opposite influence seems plausible (e.g., gratitude). In order to test such hypotheses, multiple-wave longitudinal studies are needed. It would also be informative to include measures of intelligence in future studies. Although it seems that variance in school achievement explained by personality is largely independent of the variance explained by intelligence, intelligence does play an important role in predicting school achievement, and should not be neglected. It might be especially interesting to study interactions of character strengths and intelligence in predicting academic outcomes.

Both types of teacher ratings that we used to measure positive classroom behavior encompass aspects of positive achievement-related behavior (e.g., behaving diligently) as well as positive social behavior (e.g., showing appropriate conflict management). These two aspects are not clearly separable in the ratings that were used here, and factor analyses clearly suggested a one-factor-solution. This may also be due to the fact that the majority of the items covered achievement-related behavior. However, it might be informative to further develop those ratings to measure the two aspects separately and better understand whether positive classroom behavior is indeed unidimensional or whether it can also be conceptualized in a multidimensional way. With a multidimensional assessment of positive classroom behavior, perhaps additional strengths could emerge as predictors or as stronger predictors of positive classroom behavior.

Similarly, other types of academic outcomes besides grades might be investigated in future studies. For instance, results by Kappe and van der Flier (2010) revealed that the predictive validity of the Big Five personality factors on academic performance varied to some extent with the type of academic outcome (i.e., grade, exam result, essay, team project, or thesis) considered. We would expect certain character strengths to be more strongly related with specific types of academic outcomes than others (e.g., other-directed strengths such as teamwork or fairness should be more strongly related to performance in team projects than in exams).

We also believe that studying the relationship of character strengths with other desired and important outcomes in the classroom, such as positive relationships with teachers and with peers, deserves more empirical attention (cf. Quinlan et al., 2015). For a number of character strengths, we speculated that positive relationships in the classroom might be mechanisms by which they might influence behavior and success at school. A promising direction for further research might be to contrast different potential mediators to understand the effects of different character strengths in and outside the classroom better. Our results underline the importance of positive behavior in the classroom as a mediator, but for many of character strengths the effect on school achievement was not completely or at all attributable to differences in positive classroom behavior (e.g., perspective, leadership, and humor in Study 1, and love of learning, perspective, zest, forgiveness, gratitude, and hope in Study 2). Weber et al. (2014) suggested school-related positive affect as a mediator between certain affect-favoring character strengths (zest, perseverance, love of learning, social intelligence), positive school functioning, and school achievement. Including such dimensions of positive experiences, together with variables on the relationships in the classroom, variables assessing cognitive and motivational processes (e.g., achievement goals), and positive classroom behavior, could help determine which are the most relevant mechanisms of each of the character strengths associated with school achievement.

## Conclusion

Taken together, results of the two studies reported here and in previous studies (Weber and Ruch, 2012) suggest a rather distinct set of strengths that seem to be most relevant in school. We found it interesting that these are not part of the same factor nor belong to the same virtue. In fact, strengths from four of the five factors reported in Ruch et al. (2014b) were among those consistently correlated with school achievement, positive classroom behavior, or both. However, the present findings hint at the existence of differences in the composition of this set of strengths, depending on the age, the school type, and also the type of outcome studied. Those moderators are not well understood yet. Additionally, an interesting direction for future research would be investigating the application of different character strengths in the classroom. Especially since many interventions build on the application of signature strengths, it would be interesting to see whether findings on the application of character strengths in the workplace (cf. Harzer and Ruch, 2013) would generalize to the classroom. A first question would be whether those strengths that yield relationships with desired classroom outcomes such as school achievement are also perceived to be most desirable at school by both students and teachers. Second, it would be interesting to study whether the number of signature strengths a student applies in school is also associated with satisfaction and achievement at school. It is an ongoing debate whether interventions should rather target specific strengths that are seen as most relevant in the school context, or whether they should encourage the identification and application of the individual student’s set of signature strengths (cf. Linkins et al., 2015), and potentially also encourage schools to provide opportunities to apply strengths that are not usually seen as relevant for school. In any case, this would have important implications for strength-based interventions.

### Conflict of Interest Statement

The authors declare that the research was conducted in the absence of any commercial or financial relationships that could be construed as a potential conflict of interest.
